# Structural and Magnetic Properties of Ni_0.8_Fe_0.2_/Ti Nanoscale Multilayers

**DOI:** 10.3390/nano8100780

**Published:** 2018-09-30

**Authors:** Ricardo López Antón, Juan A. González, Juan P. Andrés, Andrei V. Svalov, Galina V. Kurlyandskaya

**Affiliations:** 1 Instituto Regional de Investigación Científica Aplicada (IRICA) and Departamento de Física Aplicada, Universidad de Castilla-La Mancha, 13071 Ciudad Real, Spain; j.a.gonzalez@uclm.es (J.A.G.); juanpedro.andres@uclm.es (J.P.A.); 2 Department of Solid State Magnetism, Institute of Natural Sciences and Mathematics, Ural Federal University, 620002 Ekaterinburg, Russia; Andrey.svalov@urfu.ru (A.V.S.); galina@we.lc.ehu.es (G.V.K.); 3 Departamento de Electricidad y Electrónica, Universidad del País Vasco (UPV/EHU), 48080 Bilbao, Spain

**Keywords:** magnetic properties, thin films and multilayers, sputtering

## Abstract

The influence of the thickness of the Ni_0.8_Fe_0.2_ (Permalloy, Py) layers on the structural and magnetic properties of magnetron sputtered Py/Ti multilayers was studied. The thickness of the Py layers was varied in the interval of 8 to 30 Å. X-ray reflectivity scans evidence the existence of a well-defined layered structure in all the samples considered, but also the presence of a complex intermixed interface. The shape of both the temperature dependence of magnetization and the hysteresis loops of the multilayered structures depends strongly on Py thickness. Magnetic and reflectivity measurements were comparatively analyzed in order to better understand the structure of the samples, and specifically, their interfaces. In particular, the presence of small superparamagnetic Py at the interfaces of the samples, especially evident in the samples with the thinnest Py layers, seems confirmed by the magnetic measurements, agreeing well with the reflectivity results.

## 1. Introduction

In recent years, the synthesis of magnetic materials in the form of thin films and nanoparticles has been investigated for various applications due to their unique magnetic properties [1,2,3]. Magnetically soft films of Permalloy (Py) were the topic of special attention of researchers for many years, first of all, due to their wide use in micro- and nanoelectronics [4,5]. Currently, these films are the main functional material of a wide variety of devices for hard disk drives, magnetic recording devices, electronic navigation systems, position detection, object motion, and magnetic biosensors [6,7,8], as well as new promising spintronic devices (e.g., film storage devices information with high density based on magnetic vortices [9,10]). In electronic devices, Permalloy films are typically used in multilayer film structures such as, for example, spin valves [5,8] or multilayer structures with a giant magnetoimpedance effect [11,12]. It should be also mentioned that a lot of experience has been accumulated in the use of Ti layers in microelectronics [13].

Previous studies of the structural and magnetic properties of Py/Ti multilayers indicate the presence of mutual diffusion between the layers [14]. The change in magnetic properties can be interpreted in terms of a magnetically “dead” layer. In that work, it was found that at room temperature the “dead” layer thickness at each interface was close to (7 ± 1) Å if symmetric interlayer mixing is assumed. Nevertheless, there should be a continuous change with the increase of the Ti content in the three-component phase of FeNiTi. Moreover, films prepared by the ion-plasma sputtering method may have an island structure at the initial stages of formation [15]. Thus, if the thickness of the Py layer is less than a certain value, the layers of Py/Ti multilayers can have a granular structure, i.e., they can consist on Py nanogranules embedded in a non-magnetic Ti matrix or a three-component FeNiTi phase. Similar granular structures were observed, for example, in Py/Al_2_O_3_ multilayers [16]. Knowing what is really happening at the interface between the layers of Py and Ti is important both from the point of view of GMI sensors for biodetection [8] and as a way of creating special ferromagnetic films for integrated radio-frequency passive components in radio-frequency/complementary metal-oxide semiconductor technology [17], or tuning the dynamics of magnetization in magnetic multilayers [18].

X-ray reflectivity is a powerful technique to probe the structure of multilayered samples, as it can access, nondestructively, the details of buried interfaces [19]. Hence, X-ray reflectivity, in addition to x-ray diffraction and adequate magnetic measurements, could help to enlighten how the interfaces are in this system.

In this paper, we study and correlate structural and magnetic properties of Py/Ti nanoscale multilayers with different thicknesses of the magnetic component aiming to evaluate the interface contributions.

## 2. Materials and Methods

Py/Ti nanostructured multilayers were deposited by magnetron sputtering at room temperature onto Si substrates using a Fe 20%–Ni 80% alloy target. The background pressure was 3 × 10^−7^ mbar. The deposition was performed in an Ar atmosphere with 3.8 × 10^−3^ mbar working residual pressure. The deposition rates were approximately 1 Å/s for Py and 0.7 Å/s for Ti layers. The thickness of the Py layers (t_Py_) varied in intervals from 8 Å to 30 Å, whereas the thickness of Ti spacers was 30 Å for all the samples. Each multilayered structure consisted of 16 magnetic layers separated by non-magnetic spacers. Hereafter, the samples will be referred to from their Py thickness, in Å (Py8, Py15, Py24, and Py30). A magnetic field of 250 Oe was applied during sample preparation parallel to the substrate surface in order to induce a uniaxial magnetic anisotropy. X-ray reflectivity measurements were taken in a conventional θ-θ reflectometer equipped with a Göbel mirror, a knife edge filter, and CuK_α_ radiation.

Magnetic measurements were performed using an EverCool MPMS-XL SQUID magnetometer (Quantum Design, San Diego, CA, USA) for the in-plane geometry. The hysteresis loops were obtained at different temperatures with an applied field of up to 50 kOe. Field-cooled (FC) and zero-field-cooled (ZFC) magnetization curves [20] were registered (in an applied field of 100 Oe upon heating from 5 to 350 K). The diamagnetic contribution from the silicon substrates have been corrected in all the hysteresis loops shown below using pure silicon substrate calibration.

## 3. Results

### 3.1. X-ray Reflectivity

**Figure 1:**
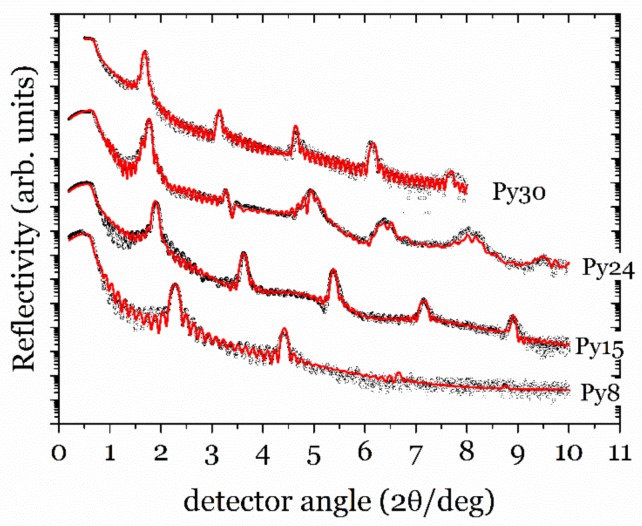
Experimental X-ray reflectivity data (open circles) and simulations (line) for all the samples.

We have conducted X-ray specular reflectivity on all the samples, which gives access to the artificial structure of the samples. The experimental scans are included as open circles in Figure 1. In this geometry, the details in the plane are averaged as the momentum transfer scans only the direction perpendicular to the sample surface (q_z_).

We have simulated and fitted the aforementioned q_z_ scans using the GenX software that implements genetic algorithms [21]. Each layer is defined by its thickness, density, and roughness, so in complex samples, such as those studied here, a considerable number of parameters come into play in the simulations. For this reason, it is important to keep the model as simple as possible. Except for the first bilayer deposited on the substrate and the upper Py layer (partially oxidized), that are modeled separately, the main block of 14 bilayers is modeled as a repetition of a single Py/Ti bilayer, whose parameters reflect the main characteristics (averaged in the block) of the sample. The densities of the layers were always kept close to the nominal values, sometimes slightly smaller as the Ar present in the sputtering chamber during the growth induces the presence of voids that reduce the density below the bulk value, especially when the growth is made at room temperature. The roughness of the layers was also quite small, only a few Angstroms, indicating the formation of a well-defined multilayered structure.

The reflectivity profile is made up from the sum of several series of peaks with different spacings coming from the different periodicities in the sample. Interestingly, the positions of the major (Bragg) peaks are determined by the main artificial periodicity of the multilayer (Λ = t_Py_ + t_Ti_), which is always obtained in the fits with great accuracy. The relative intensity of the Bragg peaks, in turn, are mainly determined by the relative thickness of Py and Ti layers [19]. In the first modeling of the structures, the total periodicity (Λ) was found to be very close to the nominal value, indicating that the calibration of the growth rates was accurate, but the fitted values of t_Ti_ and t_Py_ were, in all cases, far from the nominal values. This indicates that the interface of the two layers is probably more complex, including an intermixed region that is ascribed to one on the layers in this simplified model (see Table 1).

As this interfacial region has a tremendous effect on the magnetic properties of the multilayers, we need to characterize it in more detail. We have modified the original “bilayer” model to include interfacial layers with a mixture of Py and Ti (as will be explained below, both magnetic measurements and X-Ray diffraction also suggest that this intermixed layer exists). The modeled structure is therefore Si/Py/Ti/[Alloy/Py/Alloy/Ti]_x14_/Py/NiFe_2_O_4_. Oxidation of the upper Py layer was modeled as a thinner Py layer below an oxide layer of NiFe_2_O_4_, which seems to be most frequent in this case [22]. The two regions of alloy are kept identical to make the model simpler, but its composition and thickness are fitted independently on each sample. Table 1 shows the most relevant parameters involved in the simulations shown in Figure 1.

We have successfully simulated all the reflectivity scans with an alloy layer approximately 10 Å thick, very close to the 8 Å value that, in a previous study [14], was assigned to a dead interfacial layer. In general this alloy takes more “thickness” from the Ti than from Py layers.

### 3.2. X-ray Diffraction

**Figure 2:**
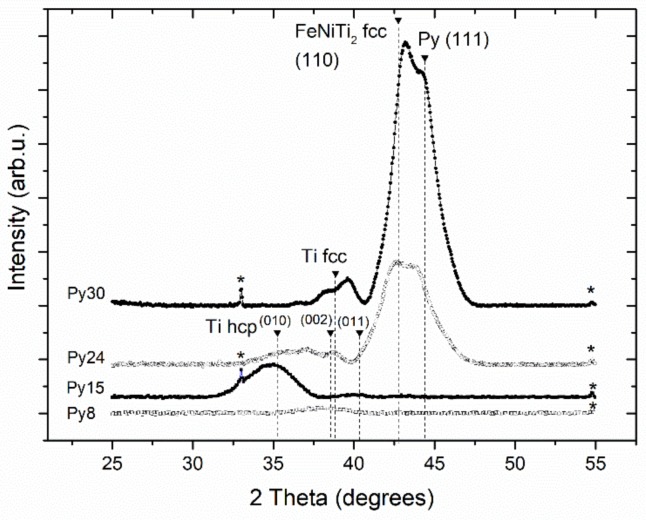
X-ray diffraction specular scans for all the samples studied. The asterisks indicate minor reflections of the Si substrate.

Figure 2 presents the X-ray diffraction (XRD) scans of all the samples studied. For the Py8 sample, the scan shows a broad peak typical of an amorphous phase around the hcp Ti [002] reflection (2θ = 38.43°), indicating that this sample is the most disordered one. As Py thickness increases to 15 Å, peaks for [010] and [011] orientations of hcp Ti appear, showing a clear change in texture in the [010] direction. As Ti thickness reaches 24 Å, two important changes occurs in the XRD scan: a double peak appears due to a crystalline Py layer, textured in the [111] direction of the cubic phase; and the existence of cubic FeNiTi_2_ textured in the [110] direction. Its formation has been previously reported in Py/Ti multilayers [14]. Regarding the Ti layer, the [010] texture disappears in benefit of the [002] direction and the possible appearance of Ti bcc. The presence of Ti bcc as well as the changes in Ti texture have been previously observed in Fe/Ti multilayers [23].

For t_Py_ above 24 Å, secondary Laue oscillations are visible on the low-angle side of the main Bragg peak associated with both crystalline FeNiTi_2_ and Py. The appearance of these peaks around the Py peak indicates a good reproducibility of its crystalline structure along the multilayer. Observing the Laue oscillations on the low-angle side and not on the high-angle side indicates that the separation of crystalline planes has expanded at the interfaces with regard to the center of the layers [24]. Such spacing differences can be explained by the interstitial diffusion of Ti in Py at the interfaces during the sputtering process [25,26]. This causes the expansion of the lattice at the Py-Ti interface, where FeNiTi_2_ is created. This effect created by the diffusion of Ti has been previously reported in similar systems such as Ti/Fe [23] and Ti/Ni [24].

It is important to remember that both specular reflectivity and diffraction are only sensible to the in-plane averaged properties of the sample. Hence, provided that the vertical profile of density is the same, the previous analysis cannot distinguish between two scenarios: (a) a solid solution alloy of Py and Ti or (b) Py particles embedded on a Ti matrix. Therefore, to gain understanding on this point, magnetic measurements were conducted.

### 3.3. Magnetic Measurements

**Figure 3:**
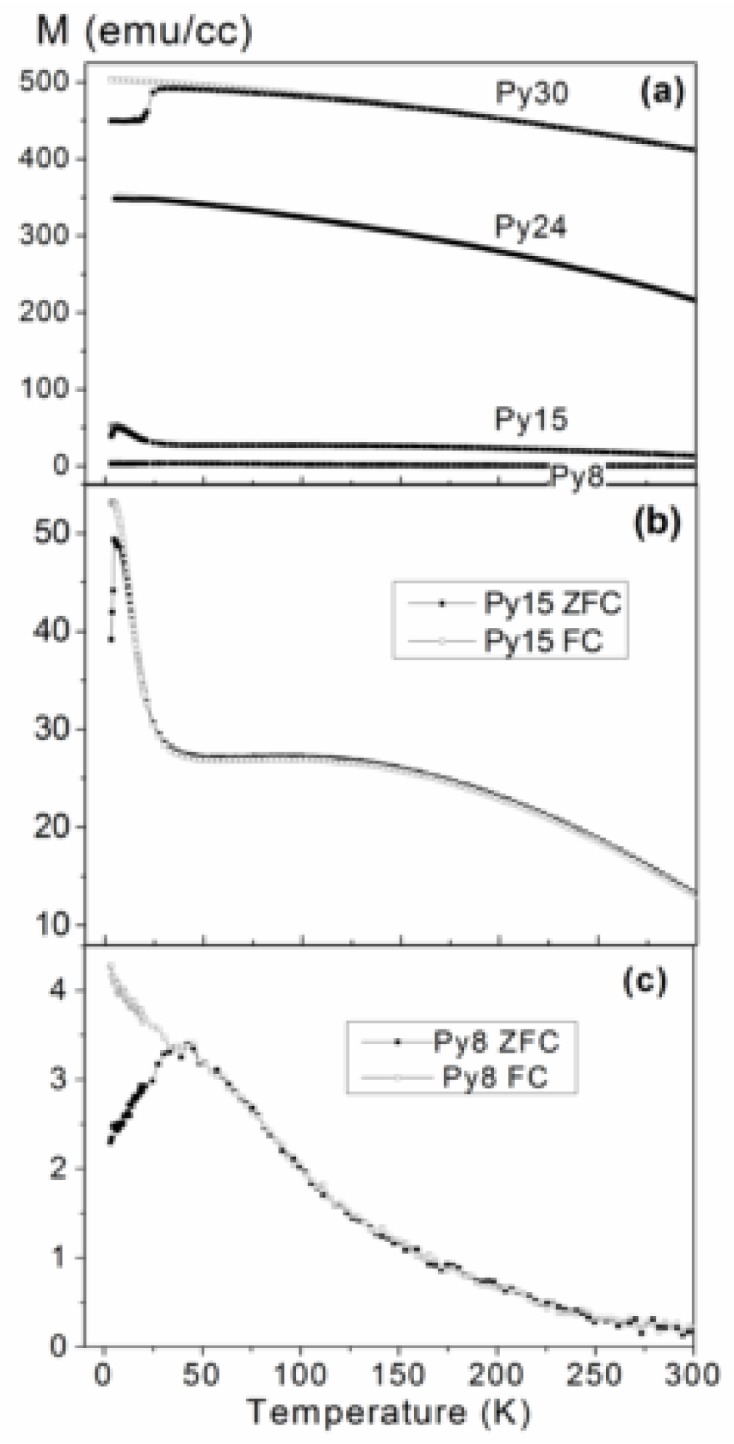
(**a**) Zero-field-cooled (ZFC) (solid squares) and field-cooled (FC) (empty squares) magnetization curves of all the samples (under an applied field of 100 Oe); detail of the ZFC-FC curves for the sample Py15 (**b**) and Py8 (**c**).

Figure 3 displays ZFC and FC magnetization curves of all the samples. As can be observed in Figure 2a, the thickest samples (Py24 and Py30) exhibit the typical shape for ferromagnetic (FM) materials (with the magnetization gently decreasing as the temperature increases) but with a certain splitting of the ZFC and FC curves at low temperature.

In the case of Py30, a sharp step in the ZFC curve is found at approximately 50 K, likely due to the formation of closing domains in the magnetic layers. Meanwhile, a peak is found in both ZFC curves of samples Py15 and Py8 (see Figure 3b,c), a sharp peak at approximately 5 K for the sample Py15, and a broad peak for the sample Py8 at approximately50 K. Those kinds of peaks in ZFC curves are usually related to the blocking temperature, T_B_, of superparamagnetic (SPM) nanoparticles (NPs). Therefore, in both samples it seems that there is a SPM phase, likely due to the mixing between Ti and Py at the interfaces. That SPM phase is likely also found in the thickest samples but its contribution is masked by the fairly higher contribution of the continuous Py layers. The condition for the superparamagnetic behavior of spherical NPs presenting uniaxial anisotropy can be expressed as [27]:

K·V = 25 k_B_T_B_, (1)

where V is the volume of the nanoparticle, K is the anisotropy energy, and k_B_ is the Boltzmann constant. We can use this expression to estimate the size of the Py SPM NPs, but the problem in that case is determining the constant anisotropy adequately, which can be fairly different in the case of thin films or NPs from the bulk value, even more so if there is some kind of alloying (as probably is our case). However, from the previous equation, it is straightforward to directly relate the T_B_ with the size of the SPM NPs (the smaller the T_B_, the smaller the size). Therefore, the SPM NPs of the Py15 would be smaller than those of the Py8 sample. This agrees fairly well with the reflectivity model: in the case of the Py8 sample, we have just an alloy layer (with just a negligible continuous Py layer) and we have bigger NPs. On the other hand, for the Py15 case, there is also a continuous Py layer (evidenced by the plateau found in the ZFC curve in Figure 3c) and, therefore, the NPs are smaller.

**Figure 4:**
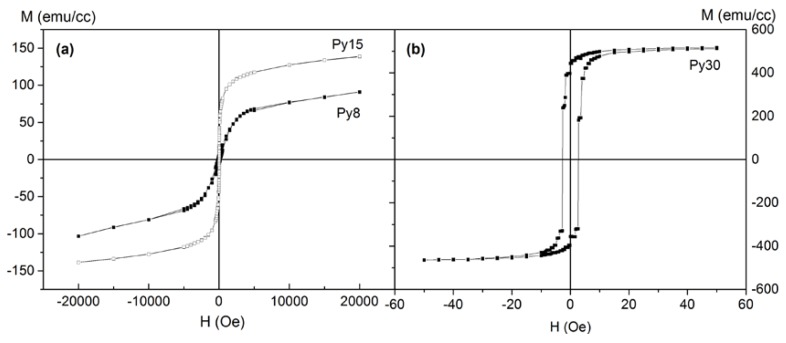
Hysteresis loops obtained at 5 K for the samples Py8 and Py15 (**a**) and for Py30 (**b**).

Figure 4 shows selected hysteresis loops measured at 5 K. In particular, the loops corresponding to the samples Py8 and Py15 are shown in Figure 4a. As can be observed in both cases, the loops do not saturate even at fields as high as 20 kOe, which is consistent with the presence of a SPM phase, as evidenced by the previous results. Meanwhile, in the case of the thicker samples, the hysteresis loop reaches saturation at very low fields, as could be expected for a continuous FM magnetic layer (as can be observed, e.g., for the sample Py30 in Figure 3b, where the contribution of SPM NPs is negligible compared to the ferromagnetism of the continuous Py layer).

**Figure 5:**
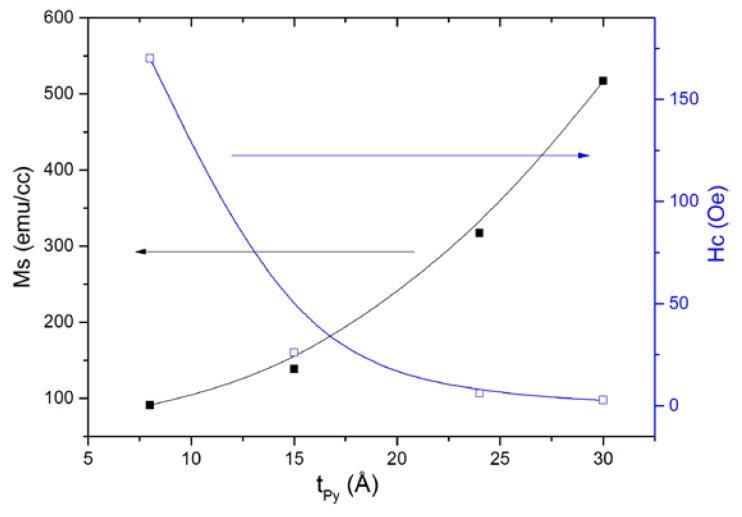
Evolution of the saturation magnetization M_S_—full squares—and coercive field H_c_—empty squares—(obtained at 5 K) versus Py thickness t_Py_. The lines are guides to the eye.

It is also noteworthy the evolution of the saturation magnetization at 5 K, Ms, (in the case of the Py8 and Py15, the magnetization at 20 kOe) and of the coercive field, H_c_, versus the nominal thickness of the Py layer-t_Py_ (see Figure 5). In particular, M_s_ decreases as the thickness is reduced but it does not disappear, as is usually assumed with a dead layer model [14]. In fact, what we observed is not a totally dead layer but instead a granular alloy of Ti and Py NPs, giving place to the SPM behavior observed in the thinner samples. On the other hand, the coercive field decreases as the thickness increases. This can explain what we have found previously: in the thickest samples, we have a continuous and thick Py layer, behaving as a soft material. For the Py15 sample, in addition to a continuous Py layer, we also have blocked SPM NPs in the alloy layer, therefore increasing the coercivity. In the Py8 sample, we have only blocked SPM NPs, and the coercive field is even higher. 

## 4. Conclusions

Py/Ti multilayers (with thickness of the Py layers between 8 and 30 Å) were successfully obtained by magnetron sputtering. The influence of the thickness of the Py layers on their structural and magnetic properties was studied, comparing structural (X-ray diffraction and reflectivity) and magnetic measurements. X-ray reflectivity measurements not only confirm the well-defined layered structure of the multilayers but also the existence of a complex intermixed interface. Both the temperature dependence of magnetization and the shape of the hysteresis loops of the multilayered structures depend strongly on layer thickness, being related to the interfaces. From the comparative analysis of the magnetic and reflectivity measurements, intermixing at the interfaces can be confirmed, as well as the appearance at the interfaces of small superparamagnetic Py nanoparticles, more evident in the thinnest samples.

## Figures and Tables

**Table 1:** Summary of relevant parameters of the X-ray reflectivity simulations for all the samples studied: nominal, fitted values, and difference between them (δ). All the quantities are in Å.

SAMPLE	Modulation (Λ)			Py Layers			Ti Layers			Alloy
	Nom.	Fitted	δΛ	Nom.	Fitted	δPy	Nom.	Fitted	δTi	Fitted
**Py8**	38	40.3	2.3	8	0.1	−7.9	30	14.5	−15.5	12.8
**Py15**	45	49.5	4.5	15	10.6	−4.4	30	19.0	−11.0	10.0
**Py24**	54	54.1	0.1	24	15.0	−9.0	30	12.7	−17.3	13.2
**Py30**	60	57.8	−2.2	30	24.6	−5.4	30	13.7	−16.3	9.8
